# Endothelial-adipocyte Cx43 Mediated Gap Junctions Can Regulate Adiposity

**DOI:** 10.1093/function/zqae029

**Published:** 2024-05-31

**Authors:** Melissa A Luse, Luke S Dunaway, Shruthi Nyshadham, Alicia Carvalho, Meghan W Sedovy, Claire A Ruddiman, Rachel Tessema, Karen Hirschi, Scott R Johnstone, Brant E Isakson

**Affiliations:** Robert M. Berne Cardiovascular Research Center, University of Virginia School of Medicine, Charlottesville, 22903, VA, USA; Department of Molecular Physiology and Biophysics, University of Virginia School of Medicine, Charlottesville, 22903, VA, USA; Robert M. Berne Cardiovascular Research Center, University of Virginia School of Medicine, Charlottesville, 22903, VA, USA; Robert M. Berne Cardiovascular Research Center, University of Virginia School of Medicine, Charlottesville, 22903, VA, USA; Robert M. Berne Cardiovascular Research Center, University of Virginia School of Medicine, Charlottesville, 22903, VA, USA; The Fralin Biomedical Research Institute at Virginia Tech Carilion, Center for Vascular and Heart Research, Roanoke, 24016, VA, USA; Robert M. Berne Cardiovascular Research Center, University of Virginia School of Medicine, Charlottesville, 22903, VA, USA; Department of Pharmacology, University of Virginia School of Medicine, Charlottesville, 22903, VA, USA; Robert M. Berne Cardiovascular Research Center, University of Virginia School of Medicine, Charlottesville, 22903, VA, USA; Department of Cell Biology, University of Virginia School of Medicine, Charlottesville, 22903, VA, USA; The Fralin Biomedical Research Institute at Virginia Tech Carilion, Center for Vascular and Heart Research, Roanoke, 24016, VA, USA; Robert M. Berne Cardiovascular Research Center, University of Virginia School of Medicine, Charlottesville, 22903, VA, USA; Department of Molecular Physiology and Biophysics, University of Virginia School of Medicine, Charlottesville, 22903, VA, USA

**Keywords:** obesity, adipose, heterocellular contact, capillaries, metabolism, adiposity

## Abstract

Obesity is a multifactorial metabolic disorder associated with endothelial dysfunction and increased risk of cardiovascular disease. Adipose capillary adipose endothelial cells (CaECs) plays a crucial role in lipid transport and storage. Here, we investigated the mechanisms underlying CaEC-adipocyte interaction and its impact on metabolic function. Single-cell RNA sequencing (scRNAseq) revealed an enrichment of fatty acid handling machinery in CaECs from high fat diet (HFD) mice, suggesting their specialized role in lipid metabolism. Transmission electron microscopy (TEM) confirmed direct heterocellular contact between CaECs and adipocytes. To model this, we created an in vitro co-culture transwell system to model the heterocellular contact observed with TEM. Contact between ECs and adipocytes in vitro led to upregulation of fatty acid binding protein 4 in response to lipid stimulation, hinting intercellular signaling may be important between ECs and adipocytes. We mined our and others scRNAseq datasets to examine which connexins may be present in adipose capillaries and adipocytes and consistently identified connexin 43 (Cx43) in mouse and humans. Genetic deletion of endothelial Cx43 resulted in increased epididymal fat pad (eWAT) adiposity and dyslipidemia in HFD mice. Consistent with this observation, phosphorylation of Cx43 at serine 368, which closes gap junctions, was increased in HFD mice and lipid-treated ECs. Mice resistant to this post-translational modification, Cx43^S368A^, were placed on an HFD and were found to have reduced eWAT adiposity and improved lipid profiles. These findings suggest Cx43-mediated heterocellular communication as a possible regulatory mechanism of adipose tissue function.

## Graphical Abstract

**Graphical Abstract:**
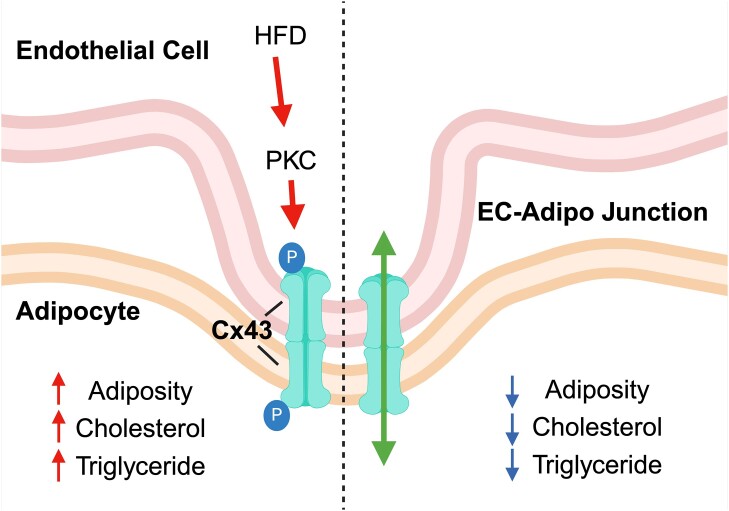

## Introduction

Adipose tissue is comprised of several cell types other than its tissue resident, adipocyte.^1^ Specifically, adipose tissue is innervated with a robust vascular network primarily made up of capillaries.^2,3^ Capillaries consist mostly of endothelial cells (ECs) and pericytes. Here, we focus on capillary adipose endothelial cells (CaECs) and the role these particular cells play in the development and progression of endothelial dysfunction in obesity.

Capillaries differ from arteries and veins in a few ways.^4,5^ One is the speed of which blood flows through the vessel. Capillaries are a low flow, low pressure environment allowing for proper gas and nutrient transfer between tissues and the blood.^6^ Specifically, capillaries in adipose tissue must take up and transport serum lipids to adipocytes for proper storage.^7^ Improper storage of lipids can result in lipotoxicity and oxidative stress both at the cellular and tissue level.^8^ Due to their location, CaECs face the majority of the lipid uptake and storage burden. To perform this specialized task, CaECs must have a unique gene expression profile for specific proteins, which will take up and transport lipids to neighboring adipocytes. The mechanism by with CaECs build this specialized store of proteins remains unknown. Does their location leave them primed to quickly respond lipid stimulation? We aim to answer these questions by looking at another way in which capillaries differ from arteries and veins, which is in their mural cell coverage. Arteries and veins are almost completely encased in a coordinated sheath of vascular smooth muscle cells (SMCs).^5^ Capillaries, however, are partially covered by pericytes,^9^ leaving ECs exposed to the interstitial space and tissue specific cells. Lack of complete coverage allows for an interesting opportunity for CaEC cell membranes to physically interact with the cell membranes of neighboring adipocytes. This is a conceivable heterocellular interaction that has not yet been extensively explored. Here, we hypothesized that CaECs are able to uniquely respond to lipid stimulation in their environment due to their heterocellular contact with adipocytes.

Heterocellular contact is often facilitated by gap junctions comprised of connexin proteins. Connexin proteins were first described in the 1980s^10^ and have been consistently described in the vasculature ever since.^11–15^ To form a functional gap junction, each cell type must contribute a connexon that come together to form a gap junction channel connecting the cytoplasm of two cells.^14^ Importantly for their function, connexin proteins are highly modified by post translational modifications.^16,17^ Phosphorylation of specific amino acids on the carboxyl-terminus of connexin 43 (Cx43) proteins can cause opening or closing of the gap junction. The opening and closing of gap junctions in response to certain stimuli can trigger various cellular signaling cascades by allowing for or blocking the passage of second messengers from one cell cytoplasm to the next.

In the vasculature specifically, connexins have been demonstrated to connect ECs with SMCs in the arterial wall of small resistance vessels.^16,17^ This heterocellular contact dictates the vasodilatory properties of the artery itself.^18^ Here, we present data demonstrating CaEC and adipocyte heterocellular contact facilitated by Cx43 mediated gap junctions can regulate epididymal white adipose tissue (eWAT) adiposity and metabolic function.

## Methods

### Single-Cell RNA Sequencing

#### Human ECs

A previously published scRNA-seq dataset from human adipose tissue was used to create these data.^19^ ECs were subsetted, using subset() function, from previously published data using clusters defined by the original authors and confirmed by our arterial, venous, capillary, and lymphatic endothelial markers. ECs from visceral adipose tissue were used for analysis for more appropriate comparison with epididymal adipose ECs.

#### Murine Adipocytes

A previously published snRNA-seq dataset from mouse epididymal adipose tissue was used to create these data.^20^ eWAT data were used to isolate out the LFD or lean adipocyte populations.

#### Murine ECs

ScRNAseq from mouse adipose endothelium has been previously described in.^3^ Briefly, the generation of single-cell indexed libraries was performed by the School of Medicine Genome Analysis and Technology Core, RRID:SCR_018883, using the 10X Genomics chromium controller platform and the Chromium Single Cell 3′ Library and Gel Bead Kit v3.1 reagent. Around 5000 cells were targeted per sample and loaded onto each well of a Chromium Single Cell G Chip to generate single cell emulsions primed for reverse transcription. After breaking the emulsion, the single-cell specific barcoded DNAs were subjected to cDNA amplification and QC on the Agilent 4200 TapeStation Instrument, using the Agilent D5000 kit. A QC run was performed on the Illumina Miseq using the nano 300Cycle kit (1.4 Million reads/run), to estimate the number of targeted cells per sample using the Cellranger 3.0.2 function. After run completion, the Binary base call (bcl) files were converted to fastq format using the Illumina bcl2fastq2 software raw reads in fastq files were mapped to the mm10 reference murine genome and assigned to individual cells by CellRanger 5.0. Data from two separate experiments representing cells from 12 mice were analyzed in RStudio (2022.07.1) with the Seurat package (4.3.0). Sequencing yielded 27 944 cells with 54 100 features. To ensure high quality data, cells were excluded if they contained less than 200 genes, more than 5000 genes, if their transcriptome was more than 5% mitochondrial encoded, and more than 5% hemoglobin beta. This resulted in a final dataset of 17 164 cells. Data were combined using SCTransform, normalized, and 3000 variable features were chosen. UMAPs were generated using 20 principal components. Clusters were generated using a resolution of 1. Non-ECs were excluded based on low expression of Pecam1 and Cdh5 as well as high expression of non-endothelial markers (Col1a1, Acta2, Cd3g, Ptprc, Ccr5, Adipoq). Dotplots were made with assay = “RNA” to capture the most appropriate comparisons between genes.

### Electron Microscopy and Image Analysis

Six male C57BL6/N mice, purchased from Taconic were fed an NC diet (5% Kcal Fat) were used between the ages of 15-20 wk. Mice were first perfused with PBS with a subsequent second perfusion with a solution consisting of 4% PFA and 0.5% glutaraldehyde in PBS. Epididymal adipose tissue was removed and placed in a 4% PFA/0.5% glutaraldehyde solution to post fix for 48 h at 4°C. Samples were then processed for electron microscopy studies by the UVA advanced microscopy facility. Grids were imaged on a Jeol 1230 for transmission electron microscopy (TEM) analysis. For TEM analysis, multiple fields of view were used for each mouse, each mouse represents an *N* value. Membranous contact was defined as any two membranes residing within 10 nm of each other. Data represented as percentage of total contact sites observed within adipose tissue.

### Transwell Co-Culture

Human adipose derived stem cells (HASCs) (Thermofisher: R7788115) were seeded (∼150 K cells per insert) on the underside a fibronectin coated transwell insert (Fisher: 07-200-170, 0.4µm pore). This was achieved through flipping the insert over before seeing the cells, allowing the cells to adhere and then flipping the insert back over. Once the HASCs adhered, the insert was flipped back over into a 6-well dish containing stem cell growth media (DMEM low glucose with glutamax: Thermofisher 10 567 014, 10% FBS, and 0.5% gentamicin: Thermofisher 15710064). HASCs were grown to over confluency and treated with adipocyte differentiation media (DMEM high glucose Thermofisher 11 965 092, 10% FBS, 1% Penicillin-Streptomycin, 0.25% 100U insulin, 0.5 m m 3-siobutyl-1-methyxanthine (IBMX), 0.25 m m Dexamethasone, 10 µmol Pioglitazone) for 4 days. On the fifth day, the media was changed to Adipocyte media containing DMEM high glucose, 10% FBS, 1% Penicillin-Streptomycin. Every other day following, media was changed to DMEM high glucose, 5% FBS, 1% Penicilin-Streptomycin. Between days 9-12 post treatment with differentiation media, the inside of the transwell insert was coated with fibronectin. The following day between 150-200 K HAMECs (Sciencell 7200). HAMECs were allowed to settle for 72 h post seeding before experiments were performed. Lipid treatment comprised of exposure to 50µm long chain fatty acids consisting of 12.5µm linoleic acid, 25µm oleic acid, and 12.5µm palmitic acid for 8 h.^21^

### Immunostaining

Transwells were either immunostained en face or via transverse sectioning. For both forms of staining, transwells were fixed using 4% PFA directly into the well. For en face staining, transwells were fixed for 20 min at room temperature. For transverse staining, inserts were left to incubate in 4% PFA overnight at 4°C with agitation. Primary antibodies and stains used for staining were Ve-Cadherin (Abcam: ab232880, 1:200), Connexin-43 (Sigma: C6219, 1:200), Phalloidin (Thermofisher: A12380, 1:1000), and Bodipy (Thermofisher 493/503, 1:1000). Transverse sections were embedded in paraffin as in.^22^ For transverse staining, Connexin-43 (Sigma: C6219, 1:200), Phospho368-Connexin-43 (Sigma: SAB4504371, 1:200), and Carbonic anhydrase (Car4) (Thermofisher: MA5-43926, 1:100) primary antibodies were used. Tissue sections were embedded in paraffin and sectioned by the UVA histology core. Phospho368-Connexin-43 (Sigma: SAB4504371, 1:200) primary antibody was used. Images were taken on an Olympus FV3000 at 60× magnification and quantified using FIJI.

### Real-Time Quantitative Polymerase Chain Reaction (PCR)

Total RNA was extracted from mouse tissues and adipocyte fractions using the Aurum Total RNA Fatty and Fibrous Tissue Extraction Kit (Biorad: #732-6870). RNA from cells was extracted using Zymo Research R1055 Quick-RNA MiniPrep Kit, Zymo Research Kit (Genesee: 11-328). RNA concentration was measured using the Nanodrop1000 spectrophotometer (Thermo Fisher). RNA was stored at −80°C before reverse transcription with SuperScript III First-Strand Synthesis system (Thermo Fisher: 18080051) using random hexamer primers on 1 μg of template RNA. Real-time quantitative PCR was performed using Taqman Gene Expression Master Mix (Thermo Fisher: 4369016) and Taqman Real-Time PCR assays in MGB-FAM for Cx43 (Mm01179639_s1), Fabp4 (Hs00609791_m1), and were normalized to β-2-microglobin/B2M in VIC-PL (Hs00364808_m1; Mm00437762_m1). Reactions were run in a CFX Real-Time Detection System (Applied BioSystems) and threshold cycle number (CT) was used as part of the 2-DDCT method to calculate fold change from control.

### Genomic Excision

DNA was extracted from lung tissue and digested using proteinase K (Bioline: BIO-37084) for genomic excision gels. A set of two primers were used, forward: GCTACTTCTTGCTTTGACTCTGATTA and reverse: GCTCACTTGATAGTCCACTCTAAGC. Non-excised Cx43 mice have a no band and Cx43 is a positive excision band at 686 bp.

### Western Blotting

Cells and tissue lysates were generated in RIPA (50 mmol/L Tris-HCL, 150 mmol/L NaCl, 5 mmol/L EDTA,1% deoxycholate, 1% Triton-X100) in PBS and pH adjusted to 7.4 supplemented with protease inhibitor cocktail (Sigma: P8340). Lysates were rocked at 4°C for 30-60 min to solubilize proteins, sonicated briefly, and centrifuged for 15 min at 1 300 G to pellet cell debris. Protein concentration was determined using the Pierce BCA method (Thermo Fisher: 23227). Total protein of 20 μg was loaded into each sample well. Samples were subjected to sodium dodecyl-sulate (SDS) gel electrophoresis using 8% or 4-12% Bis-Tris gels (Invitrogen) and transferred nitrocellulose membranes for immunoblotting. Membranes were blocked for 1 h at room temperature in a solution containing 3% BSA in Tris buffered saline, then incubated overnight at 4°C with primary antibodies against Cx43 (Sigma: C6219, 1:1000) and Phospho368-Connexin-43 (Sigma: SAB4504371, 1:100). Membranes were washed and incubated in LiCOR IR Dye secondary antibodies (1:10 000) for 1 h and viewed/quantified using the LiCOR Odyssey CLx with Image Studio software. Licor Total Protein stain was used for loading normalization. Representative western blot images have been cropped for presentation.

### Animals

Only male mice were used, 20-23 wk of age, on a C57Bl/6 genetic background. Cdh5ERT2+/Cx43fl/fl mice were cared for under the provisions of the University of Virginia Animal Care and Use Committee, while Cx43WT and Cx43S368A animals were cared for under the provisions of Virginia Tech Animal Care and Use Committee. Both facilities follow the National Institutes of Health guidelines for the care and use of laboratory animals. Animals were subject to a 12-h light dark cycle. The inducible, EC-specific Cx43 knockout mice (Cdh5ERT2+/CX43fl/fl) were generated by crossing Cdh5ERT2+/CxWT/WT mice (a kind gift from Dr Ralf Adams, Max Plank Institute, Germany) with Cdh5ERT2−/Cx43fl/fl mice.^23^ To conditionally induce Cx43 deletion in the vascular endothelium, Cdh5ERT2−/Cx43fl/fl (EC Cre− Cx43fl/fl) and Cdh5ERT2+/Cx43fl/fl (EC Cre+ Cx43fl/fl) littermates were fed tamoxifen diet (Envigo: TD130856) for two consecutive weeks starting at 6 wk old. Littermates were then fed HFD (60% Kcal from fat Bio-Serv-The Foster Corp F3282) starting at 8 wk of age for 12 consecutive weeks. For all assessments of blood, blood was collected via terminal cardiac puncture using a syringe fitted with 25 G needle, coated with ethylene glycol-bis(β-aminoethyl ether)-N,N,N′,N′-tetraacetic acid (EGTA) to prevent clotting and deposited in gold cap blood collection tubes. Blood lipids (cholesterol and triglycerides) were processed by UVA clinical laboratory.

### Statistical Analysis

Statistical analysis was performed using Prism GraphPad 9. Specific statistical test type and *N* values are listed in each corresponding figure legend. Data represented are the mean with SEM (+ and −) shown as error bars. All *N* values represent different experiments (in vitro) and individual mice (in vivo). For image quantification, three fields of view were used to average each *N* value.

## Results

### Fatty Acid Machinery Is Enriched in CaECs

**Figure 1.**
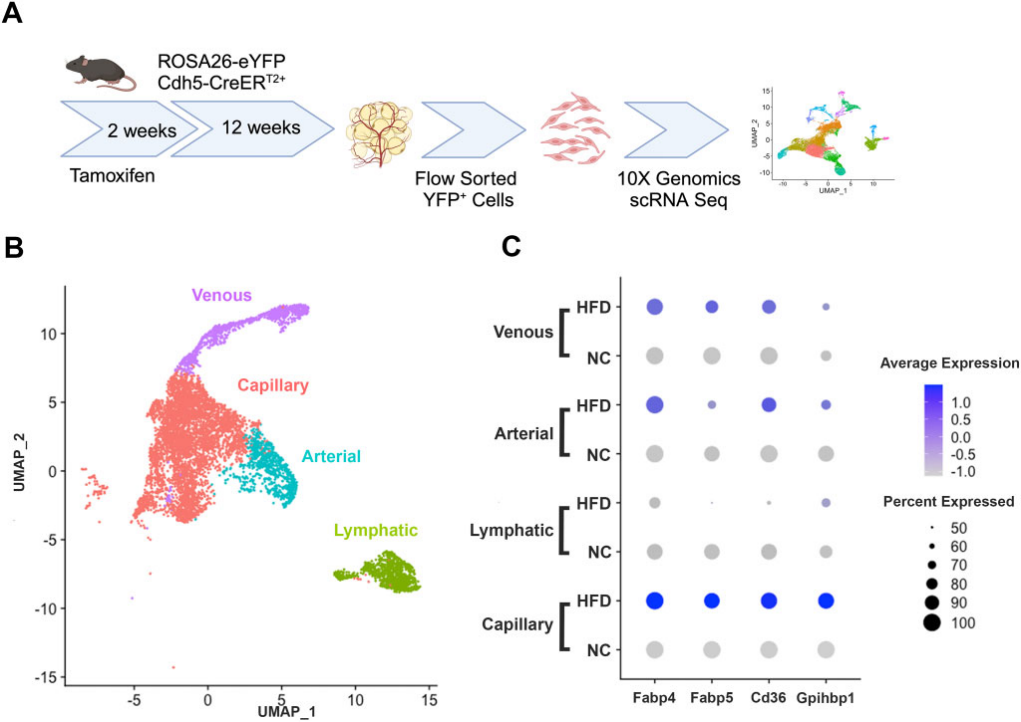
Adipose endothelial cells express fatty acid machinery during high fat diet. (A) Schematic with scRNAseq experimental design and workflow; (B) UMAP projection of adipose endothelial cells, from both NC and HFD mice, broken down by EC subtype. These clusters were defined using known markers for arterial (Efnb2, Dll4, Gja5), capillary (Apq7, Rgcc, Gpihbp1), venous (Plvap, Ackr1, Selp), and lymphatic (Prox1, Lyve1, Flt4) endothelial cells. (C) Dotplot showing expression of fatty acid uptake and transport machinery expression broken down by EC subtype and diet.

Single-cell RNA sequencing (scRNAseq) of adipose ECs from normal chow (NC) and high fat diet (HFD)-fed mice (Figure 1A) show an enrichment of fatty acid binding and transport machinery (ie, *Fabp4, Fapb5, CD36, Gpihbp1*) in capillary ECs (Figure 1B and C). This increased gene expression is specific to capillaries from HFD-fed mice demonstrating their importance in lipid handling as compared to other EC types.

### CaECs Make Contact with Adipocytes

**Figure 2.**
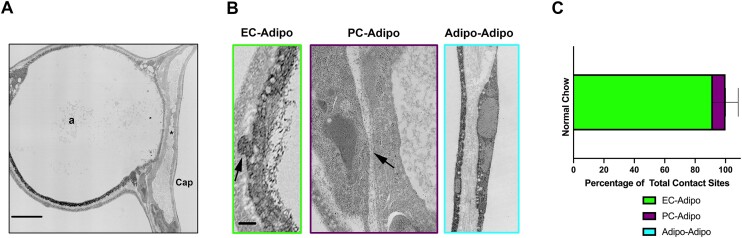
Capillary adipose endothelial cells are in direct contact with adipocytes. (A) Transmission electron micrograph (TEM) of an adipocyte (a) and capillary (cap, *denotes lumen). Scale bar denotes 2 μm. (B) TEM showing different types of cellular contact in adipose tissue. Left demonstrates endothelial (EC) and adipocyte (Adipo) contact, middle showing pericyte (PC) and adipocyte contact and right, showing lack of adipocyte-adipocyte contact. Scale bar denotes 500 nm. (C) Quantification of contact seen in adipose tissue normalized as a percentage of total contact sites seen in tissue. *N* = 4. Arrows indicate sites of contact.

**Figure 3.**
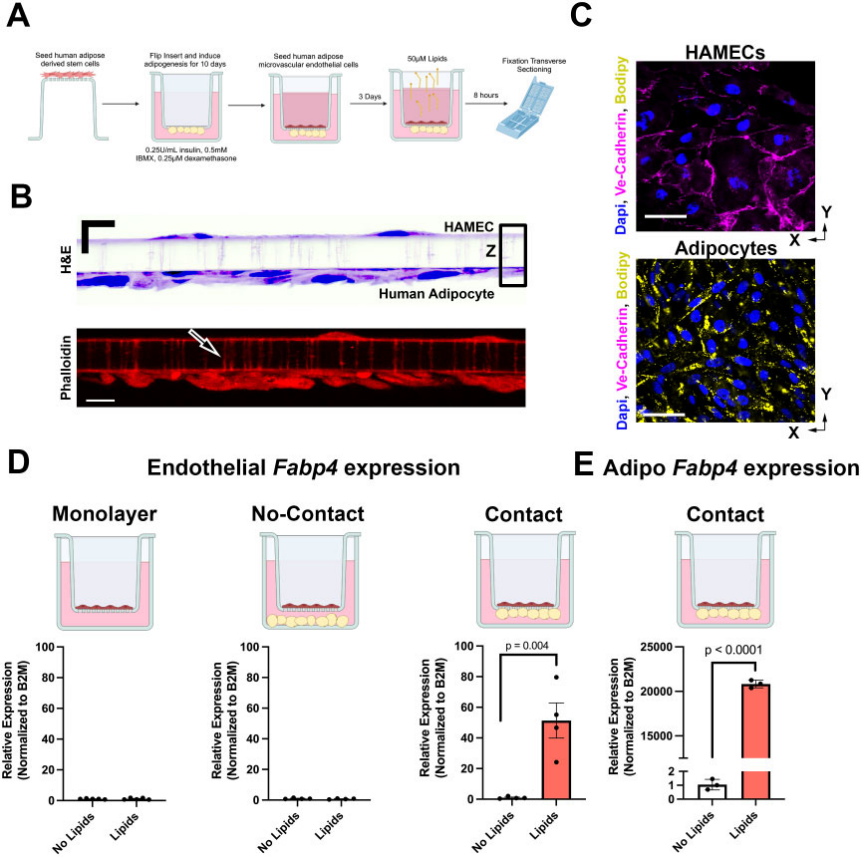
Fatty acid gene expression in adipose endothelial cells is dependent on contact with adipocytes. (A) Schematic showing experimental methods for the development of endothelial-adipocyte co-culture model using transwell inserts. (B) Transverse view of transwell insert sectioned and stained with H&E (top) and Phalloidin (bottom). Human adipose endothelial cells (HAMECs) are on the top side of the transwell with differentiated human adipocytes on the bottom. Arrow is pointing to cellular contents (marked by phalloidin) in the pores of the transwell. Scale bar represents 10 μm. (C) En face view of the transwell insert. HAMECs shown in top image staining with EC marker Ve-Cadherin (magenta: top image) and adipocytes from the underside of the same transwell are shown below with robust lipid droplet staining (Bodipy—yellow: bottom image). Scale bar represents 20 μm. (D) Fatty acid binding protein (Fabp4) expression in HAMECs from monolayer (left) no-contact co-culture (middle) and contact (right) co-culture models after 50 μm lipid treatment of HAMECs 50 μm long chain fatty acids (consisting of 12.5 μm linoleic acid, 25 μm Oleic acid, and 12.5 μM Palmitic acid for 8 h) *N* = 4-5. (E) Adipocyte Fabp4 expression from contact co-culture after lipid stimulation of HAMEC layer, *N* = 3. Statistics represent Student’s *t*-test.

To understand why this gene expression change is specific to capillary endothelium, we examined the ultrastructure of epididymal adipose tissue capillaries (Figure 2A). In several images, we found significant instances of cellular contact between CaECs and adipocytes (Figure 2B, left). A different form of cellular contact seen in adipose tissue was between pericytes and adipocytes (Figure 2B, middle). In all sections of tissue examined, there were no instances of adipocyte to adipocyte cellular contact observed (Figure 2B right). Interestingly, only the CaEC and adipocyte contact sites had the ball-and-socket structure reminiscent of myoendothelial junctions (MEJs) between endothelium and smooth muscle in arteries (eg, ^11^,^24–27^). These contact sites were quantified as a percentage of total contact between cells observed within adipose tissue (Figure 2C). Based on these data, we have found CaEC to adipocyte contact makes up the majority of heterocellular contact observed in adipose tissue. To model this physical interaction in vitro, we developed a co-culture model using transwell inserts with pores to allow for cellular projections to occur (Figure 3A). Using our co-culture model, the transwell insert can be sectioned transversely (Figure 3B) to examine the morphology (hematoxylin and eosin; H&E) and cellular projections (Phalloidin) of the human adipose microvascular ECs (HAMECs) and differentiated human adipocytes. The transwell inserts can also be immuno-stained en face to look at both the top HAMEC layer and the bottom adipocyte layer. We show HAMECs (Figure 3C, top) with robust staining of vascular marker Ve-Cadherin and almost no lipid droplets. Additionally, the bottom adipocyte layer (Figure 3C, bottom) has robust lipid droplet formation, a hallmark of mature adipocytes. Once we developed a working co-culture model where heterocellular contacts are made, we were able to mechanistically probe how heterocellular contact can affect gene expression. To recapitulate the fatty acid gene expression seen in Figure 1C, we used *Fabp4* expression as a reporter for lipid stimulation. When HAMECs in monolayer (Figure 3D, left) or HAMECs grown in no-contact co-culture (Figure 3D, middle) were stimulated with lipids, there was no detectable *Fabp4* expression. HAMECs grown in contact co-culture with adipocytes upregulated *Fabp4* significantly when treated with lipids. Adipocytes also increased *Fabp4* expression when in contact co-culture with HAMECs exposed to lipids (Figure 3E).

### Cx43 May Regulate Capillary Adipose Endothelial and Adipocyte Junctions

**Figure 4.**
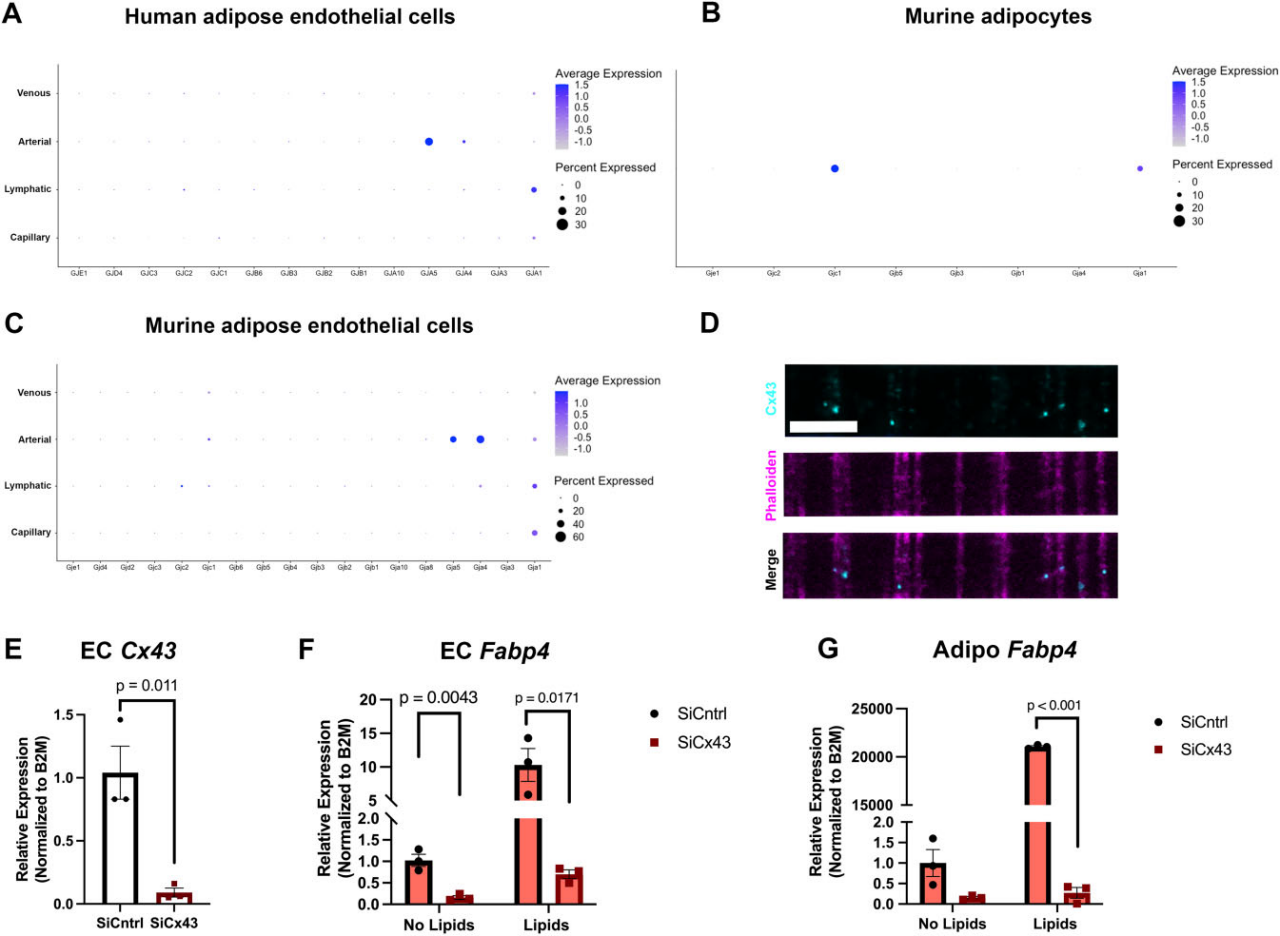
Cx43 is expressed in capillary endothelial cells and adipocytes. (A) Dotplot of all connexins expressed in human adipose endothelial cells divided by EC subtype. Connexin43 is shown with gene name as GJA1. (B) Dotplot of all connexins expressed in murine adipocytes. (C) Dotplot of all connexins expressed in murine adipose endothelial cells divided by EC subtype. (D) Transverse section of contact co-culture model with Cx43 staining (top) and phalloidin staining (middle) and the overlap (bottom) in the pores of the transwell. Scale bar denotes 10 μm. (E) Endothelial cell (EC) Cx43 expression with siRNA knockdown of Cx43, (F) EC Fabp4 expression with or without siCx43, (G) Adipocyte (Adipo) Fabp4 expression with or without EC siCx43. Statistics represent Student’s *t*-test. Long chain fatty acids of 50 μm were used for lipid treatments (12.5 μm linoleic acid, 25 μm oleic acid, and 12.5 μm palmitic acid for 8 h).

Heterocellular contact between CaECs and adipocytes is important for fatty acid gene regulation. Gap junctions are major regulators of heterocellular contact, especially in the vasculature.^14,28^ Using scRNAseq data from human adipose ECs (Figure 4A), the only gap junctional protein detected in capillaries was connexin43 (Cx43-GJA1). To form a functional gap junction between cells via connexin proteins, each cell type contributes half the junction. We also found Cx43 to be one of the main connexins expressed in murine adipocytes (Figure 4B). Complimentary to this data, scRNAseq of murine adipose ECs mimics the data shown from human ECs in that Cx43 is the only connexin expressed in the capillary adipose endothelium (Figure 4C). The overall connexin expression of murine adipose endothelium closely resembles overall connexin expression in the mesenteric endothelium, regardless of vascular bed (Supplementary Figure S1). Given this data, we wanted to utilize our transwell co-culture system to look for Cx43 protein. We observed Cx43 puncta overlap with the cellular projections made between HAMECs and adipocytes (Figure 4D). To further understand the functional relevance of these gap junctions, we selectively knocked down Cx43 from HAMECs (Figure 4E) and demonstrated a loss of Fabp4 gene expression in endothelium (Figure 4F) and adipocytes (Figure 4G), reminiscent of a lack of cellular contact in Figure 3D. Thus, the presence of gap junctions between endothelium and adipocytes may be an important regulator of adipose function.

**Figure 5.**
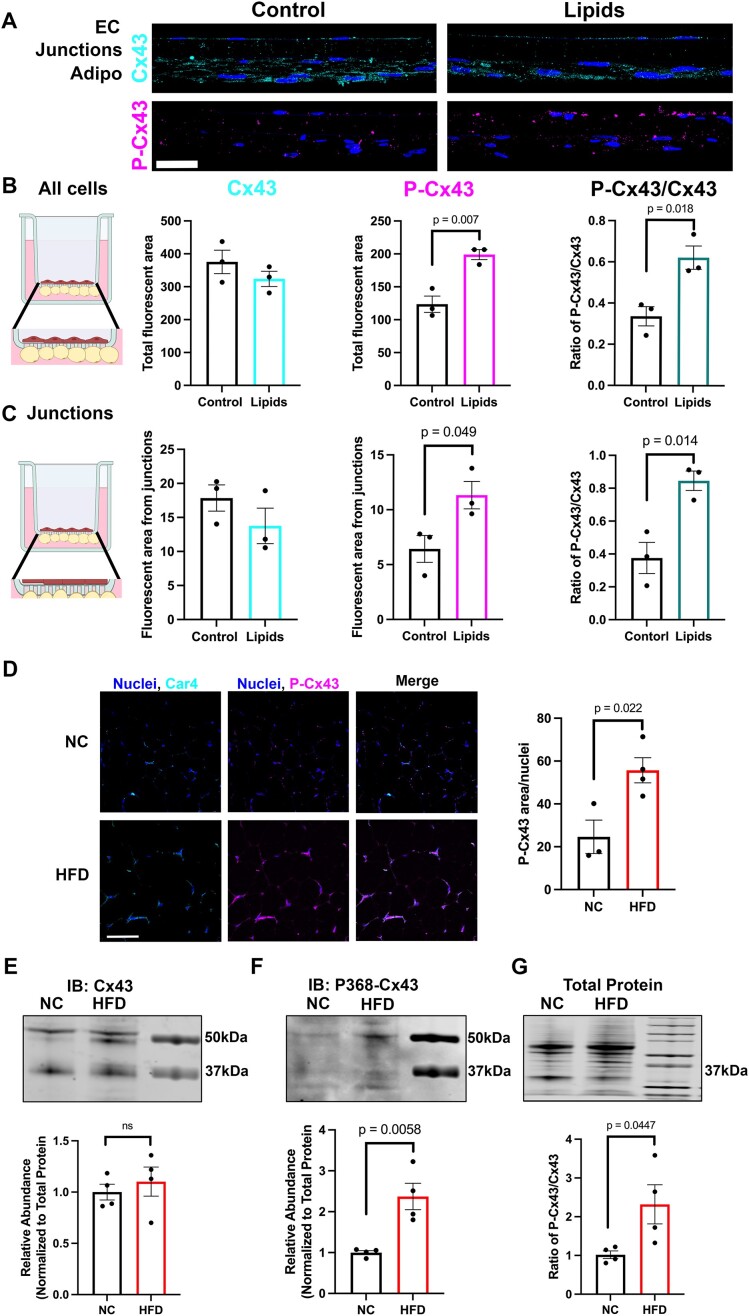
Phosphorylation of Connexin43 increases with lipid stimulation and high fat diet. (A) Transverse section of contact co-culture transwell from control or lipid-treated human adipose endothelial cells (HAMECs). The top layer of cells are HAMECs, the middle section is the pores of the transwell, and the bottom contains human adipocytes. Connexin43 (Cx43) staining is shown in cyan and phosphorylated connexin43 (P-Cx43) is shown in magenta. Scale bar represents 10 μm. Lipid-treated cells were dosed with 50 μm long chain fatty acids consisting of 12.5 μm linoleic acid, 25 μm oleic acid, and 12.5 μm palmitic acid for 8 h. (B) Quantification of P-Cx43 and Cx43 from all cells in the transverse section and (C) showing the quantification of just the junctional portion of the contact co-culture transverse section. (D) Immunofluorescence staining of epididymal fat pad sections for Car4 (capillary marker) and P368-Cx43 in NC (top) and HFD mice (bottom). Quantification of P368-Cx43 fluorescence area normalized to number of nuclei (far right). Scale bar represents 100 μm western blot of adipose tissue from normal chow (NC) and high fat diet (HFD) fed mice immunoblotting for Cx43 (E) P-Cx43 (F). Total protein is shown in (G) with ratio of P-Cx43/Cx43 signal quantified below. Right most lanes are molecular weight ladders in E-G. Statistics represent Student’s *t*-test. Data points represent individual mice (D-G).

Connexin proteins are highly regulated by post-translational modifications. These modifications can dictate the channels’ open and closed state as well as its localization.^16,17,29^ Specifically, phosphorylation of Cx43 at serine 368 can increase the closing of the Cx43 channel.^30,31^ To further investigate how Cx43 may regulate heterocellular communication between CaECs and adipocytes, we looked for phosphorylated Cx43 specifically at serine 368 (P-Cx43) using transverse sections from our transwell co-culture model. Representative immune-staining images (Figure 5A) show robust Cx43 staining in HAMECs and adipocytes in both control and lipid-treated conditions. However, upon lipid treatment, phosphorylation of Cx43 increases when quantified from all cells in Figure 5B. Additionally, we quantified P-Cx43 specifically from the portion of the transwell containing the cellular junctions and saw the same increase in P-Cx43 but not Cx43 itself (Figure 5C). To test if P-Cx43 increases via lipid stimulation in vivo, we looked specifically for in adipose tissue from mice fed a HFD for 12 wk using immunofluorescence on epididymal fat pad sections (Figure 5D). P-Cx43 was increased in adipose tissue from mice fed a HFD (Figure 5D). Capillary endothelium was marked using Car4.^4,32^ Additionally, we assessed total Cx43 protein abundance, which did not change between NC and HFD-fed mice (Figure 5E). However, P-Cx43 increased in HFD-fed animals (Figure 5F) mimicking the results we see in vitro. The ratio of phosphorylated Cx43 and total Cx43 was quantified and normalized to total protein (Figure 5G). These data show Cx43 is present at the junctions between CaECs and adipocytes and phosphorylation at serine 368 is increased in HFD and may regulate heterocellular communication.

### Endothelial Cx43 Can Regulate Ewat Adiposity

**Figure 6.**
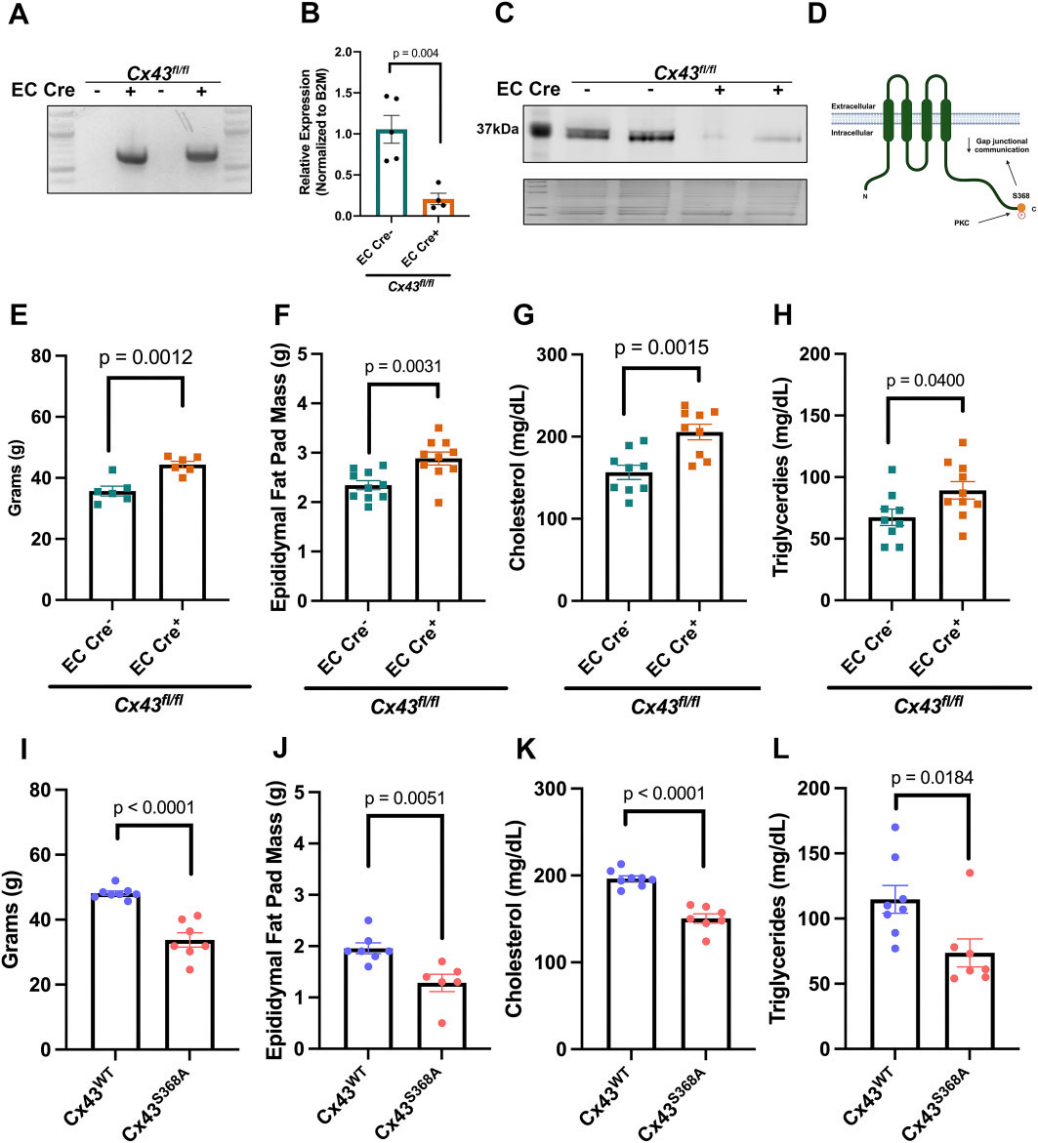
Endothelial Connexin43 can regulate eWAT fat mass and serum lipids. (A) Genomic excision of connexin43 (Cx43) showing positive excision bands. Loss of Cx43 RNA (B) and protein (C) with EC Cre induction diaphragm was used for figures A-C. Left most lane in (C) represents molecular weight markers. (D) Schematic of Connexin43 gap junction. (E) Weight gain, (F) Epididymal fat (eWAT) pad mass, (G) cholesterol, and (H) triglycerides from high fat diet (HFD) fed EC Cre- and EC Cre + Cx43fl/fl mice, *N* = 7-10. Body mass (I), epididymal fat (eWAT) pad mass (J), cholesterol (K), and triglycerides (L) from HFD-fed Cx43S368A mutant mice, *N* = 7-10. Statistics represent Student’s *t*-test.

To test our hypothesis that EC Cx43 may regulate intercellular communication between CaECs and adipocytes, we selectively deleted Cx43 from endothelium using an inducible Cdh5-Cre^ERT2+^  *Cx43^fl/fl^* mouse. Successful deletion is shown via excision of *Cx43* (Figure 6A) and reduction in both Cx43 RNA and protein (Figure 6B-C). By removing Cx43 from endothelium, we can model the gap junction being closed in vivo. As a metabolic challenge, mice were fed a HFD for 12-wk. Deletion of Cx43 from ECs caused an increase in both weight gain (Figure 6E) and eWAT fat pad mass (Figure 6F). Additionally, both serum cholesterol (Figure 6G) and triglycerides (Figure 6H) were also increased significantly in mice lacking Cx43 in endothelium. Age matched mice were also fed NC diet, however, no changes in body mass, eWAT mass, or triglycerides were observed (Supplementary Figure S2) regardless of genotype. Interestingly, cholesterol levels in NC EC Cre^+^  *Cx43^fl/fl^* were slightly elevated compared to NC fed controls. Female EC Cre^+^  *Cx43^fl/fl^* mice, fed both NC or HFD, generally demonstrated a similar trend to males (Supplementary Figure S3), but with significantly lower weights and lipid parameters due to decreased lean mass (eg, ^33^) Additionally, loss of EC Cx43 had no effect on insulin or glucose sensitivity in both NC and HFD-fed mice (Supplementary Figure S4A-D). We next examined both epididymal adipose and liver histology to examine tissue morphology (Supplementary Figure S5A-B). As expected, given the lack of glucose and insulin phenotype, there were no differences observed in overall morphology of adipose tissue (Supplementary Figure S5A). However, liver sections from HFD EC Cre^+^  *Cx43^fl/fl^* appeared to contain more lipids (Supplementary Figure S5B). Given these data, we performed Oil Red O staining to assess liver lipid content, quantification of which shows a slight increase in lipid accumulation within the liver (Supplementary Figure S5C). The liver contributes significantly to the non-esterified fatty acid (NEFA) concentrations within the blood, therefore, we examined serum NEFA levels in both NC and HFD-fed mice. Regardless of diet EC Cre^+^  *Cx43^fl/fl^* mice had increased NEFA levels (Supplementary Figure S4E-F) indicating an imbalance in lipid homeostasis. These data demonstrate an important role for EC Cx43 in the regulation of serum lipids especially in HFD.

Loss of EC Cx43 is a mimetic for the channel in a closed state; thus, to test a scenario where Cx43 gap junctions would be more likely to be open, we used a mutant mouse where serine 368 is mutated to an alanine (Cx43^S368A^). Therefore, Cx43^S368A^ (channel open) mice would be expected to have the opposite phenotypic parameters to the EC Cre^+^  *Cx43^fl/fl^* mice that have no Cx43 in endothelium (channel closed). Indeed, Cx43^S368A^ mice fed a HFD for 12 wk have decreased body weight (Figure 6I) and decreased eWAT mass (Figure 6J) compared to wildtype controls (Cx43^WT^). Furthermore, Cx43^S368A^ mice have decreased cholesterol (Figure 6K) and triglycerides (Figure 6L) in response to HFD challenge.

## Discussion

Adiposity and serum lipids are an important indicator of metabolic health and risk for cardiovascular disease.^34^ Here, we present a novel mechanism by which CaECs can regulate eWAT adiposity and serum lipids through direct contact with adipocytes. This contact is facilitated by gap junctional protein Connexin43 (Cx43), and phosphorylation of Cx43 at serine 368 regulates heterocellular communication between ECs and adipocytes. Phosphorylation, and therefore closure of the Cx43 gap junction, of Cx43 specifically at serine 368 is increased with lipid treatment and HFD. This increased channel phosphorylation and decreased EC to adipocyte communication, leading to dyslipidemia could be a potential mechanism behind the onset endothelial dysfunction in obesity.

Our scRNAseq data show an enrichment of fatty acid handling machinery (ie, *Fabp4, Fapb5, Cx36, Gpihbp1*), specifically in capillaries in response to HFD. This upregulation specifically CaECs is logical as serum lipids must pass through capillaries in adipose tissue for proper storage in adipocytes. Therefore, CaECs see a higher concentration of lipids than other endothelial beds and must increase fatty acid transport and storage proteins. Our main question based on this data was how do CaECs know their function? What about their environment within adipose tissue allows for this specific and targeted gene expression change. What is unique about capillaries is their lack of complete mural cell coverage compared to arteries and veins that have vascular SMCs.^5^ Less coverage by pericytes allows for contact between CaECs and adipocytes. Therefore, we hypothesized contact with adipocytes and exposure to high lipid levels primes CaECs to take upregulate fatty acid machinery in response to HFD.

Here, for the first time to best of our knowledge, we describe and characterize CaEC and adipocyte heterocellular contact. Heterocellular contact within the vasculature has been well described in arteries.^16–18^ In fact, the projections and contact sites we observed in adipose tissue between CaECs and adipocytes resembles the contact seen between arterial ECs and SMCs of small resistance arteries. These contact points deemed MEJs^11^ are key regulators of vasodilation and therefore systemic blood pressure.^13,18^ Therefore, it is likely the adipose-endothelial junction could regulate adipose tissue function in a similar manner to the MEJ. We demonstrate the majority of heterocellular contact in adipose tissue is between ECs and adipocytes. This positions CaECs as a window between the adipocyte and the blood, serving as an interface to relay critical information on nutrient status. This form of rapid and specific communication likely becomes crucial in times of metabolic distress, ie, HFD/obesity.

We show evidence for the importance of heterocellular contact with lipid stimulation using our co-culture transwell model. Only when in direct contact with adipocytes did human adipose microvascular endothelial cells (HAMECs) increase *Fabp4* expression. Additionally, when in direct physical contact, treating HAMECs with lipids induced a robust increase in adipocyte *Fabp4*. This physical coupling primes the adipocyte for its task of lipid uptake and storage. Proper storage of lipids is crucial to prevent lipotoxicity and the subsequent oxidative stress that follows. Data presented here show HFD increases Cx43 phosphorylation. This phosphorylation occurs specifically at serine 368 and serves as a signal for channel closure, decreasing the communication between CaECs and adipocytes. We hypothesize, this diminished communication between CaECs and adipocytes could contribute to the endothelial and adipocyte dysfunction seen in obesity.

By using mice which lack EC Cx43 (mimetic of channel closure) or express a version of the channel that is more likely to be open, we can test the metabolic effects of decreased communication between CaECs and adipocytes. Mice lacking Cx43 had worse metabolic outcomes when eating an obesogenic diet compared to mice with increased channel opening. Decreased communication between capillaries and adipocytes regarding lipid uptake and storage could account for the increase in adipose tissue mass seen in mice without EC Cx43. Additionally, metabolic disruption can result in dyslipidemia, which is also seen in mice with an EC Cx43 deletion. Increasing the channels probability of being open through the mutation of serine 368 to an alanine protected mice from metabolic dysfunction associated with HFD. Taken together, our data points to EC Cx43 as a regulator of eWAT adiposity possibly through heterocellular communication between CaECs and adipocytes.

Our data show a greater reliance on EC Cx43 communication during metabolic stress (ie, HFD feeding). We see an increase in total body mass, eWAT mass, cholesterol, and triglycerides with loss of EC Cx43 in HFD. However, mice lacking EC Cx43, which received NC diet, only have a slightly elevated serum cholesterol level. It is possible caECs and adipocytes rely on different communication pathways during homeostatic and high fat conditions. These data suggest that under metabolic stress adipocytes and ECs rely more heavily on Cx43-mediated communication.

In addition to the presence of other caEC-adipocyte signaling axis, it is possible there are additional tissues besides adipose contributing to the phenotypes seen here. For example, liver, skeletal muscle, and brown adipose are also crucial for lipid homeostasis. Here, we show a slight increase in liver lipid accumulation, it is possible skeletal muscle and brown adipose taken from loss of EC Cx43 mice could display a similar phenotype. Tissue lipid accumulation is likely due to the disruptions in lipid regulation which permeate systemically. The liver, skeletal muscle, and brown adipose are also highly vascularized specifically with capillaries. Looking for potential sites of EC contact with hepatocytes, myocytes, and brown adipocytes could further our understanding of how ECs can regulate metabolism.

Our work is not without alternative explanation. For example, we do not examine or quantify immune cell presence or contact in adipose tissue. Immune cells have been identified as mediators of adipose tissue function and health.^35^ However, the experimental method we chose to identify contact sites, TEM, is not adequate to identify and characterize contact between immune cells and adipocytes. We do not discount that immune cells, especially in HFD, can affect the proper function of adipose tissue. Additionally, we did not identify a signaling molecule, which could potentially pass between cells through Cx43 gap junctions relaying metabolic information. A number of signaling molecules have been identified throughout the literature as passing through these channels and serving as second messengers.^36–39^ Similarly, we do not directly measure, via patch clamp or dye transfer, the open and closed states of Cx43 gap junctions when phosphorylated or mutated (S368A). However, these experiments have been previously done.^40,41^ Developing therapeutic targets that could toggle the metabolic state of ECs with Cx43 activators and inhibitors could serve as novel mechanism to treat disease. This is an excellent area for future study, which could lead to more targeted methods for weight management in obese patients. Lastly, we do not discount the contributions of extracellular vesicles (EVs) passed between ECs and adipocytes in the regulation of adipose tissue metabolism.^42^ Our no contact transwell co-culture model indicates the contribution of EVs in our system is minimal as the presence of adipocytes alone was not enough to elicit *Fabp4* expression when ECs were treated with lipids. Instead, we explore an alternative mechanism for EC and adipocyte communication that could act in conjunction with these previously established pathways.

## Supplementary Material

zqae029_Supplemental_File

## Data Availability

The data underlying this article will be shared on reasonable request to the corresponding author.
