# Supplementary material for: Endothelial-adipocyte Cx43 Mediated Gap Junctions Can Regulate Adiposity
Source: Function (Oxf). 2024 May 31;5(5):zqae029. doi: 10.1093/function/zqae029 (PMC11384900; doi:10.1093/function/zqae029)
Supplement: zqae029_Supplemental_File [file zqae029_supplemental_file.docx]

**
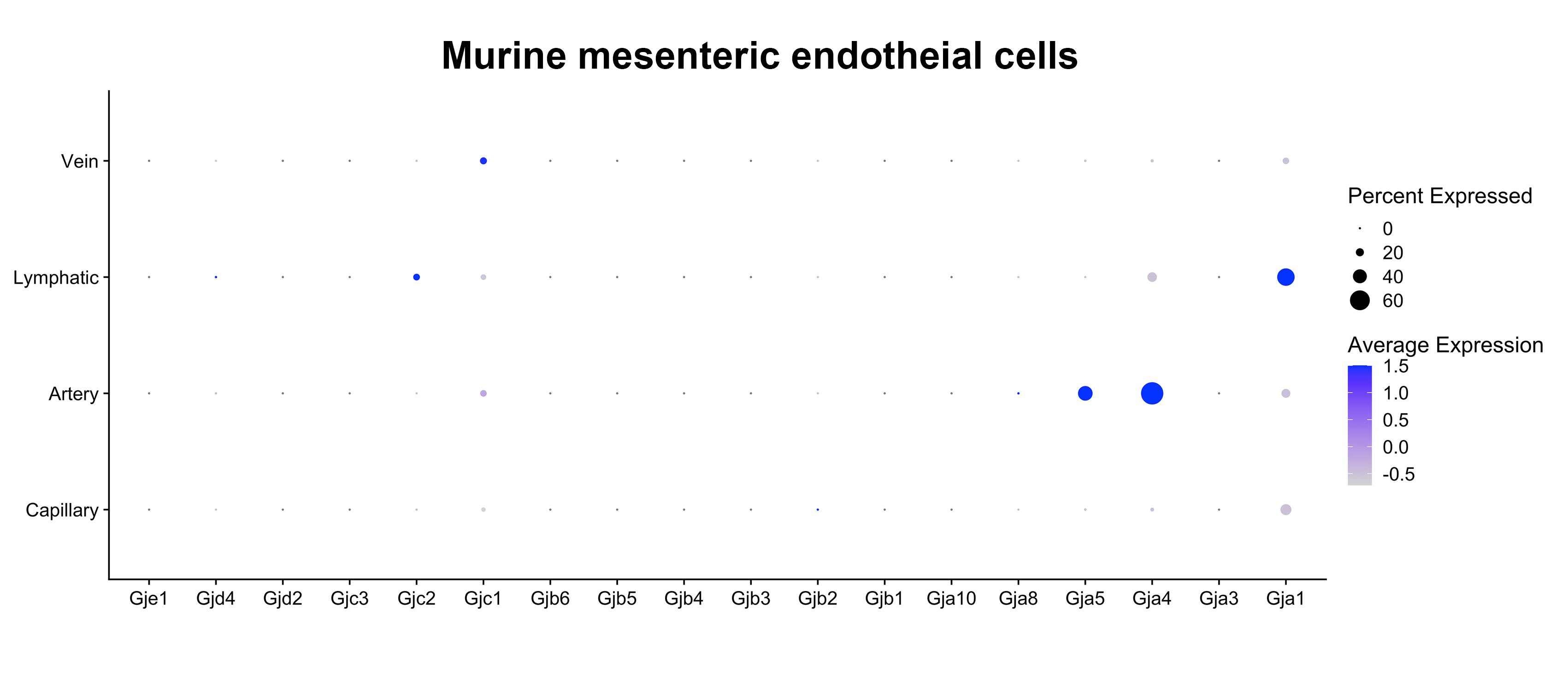
**

**Supplemental Figure 1: Connexin expression in murine mesenteric endothelial cells.** Connexin expression from murine mesenteric ECs split by vascular type (i.e. Vein, lymphatic, artery, and capillary). Raw data and vascular classification criteria mined from^3^.

**
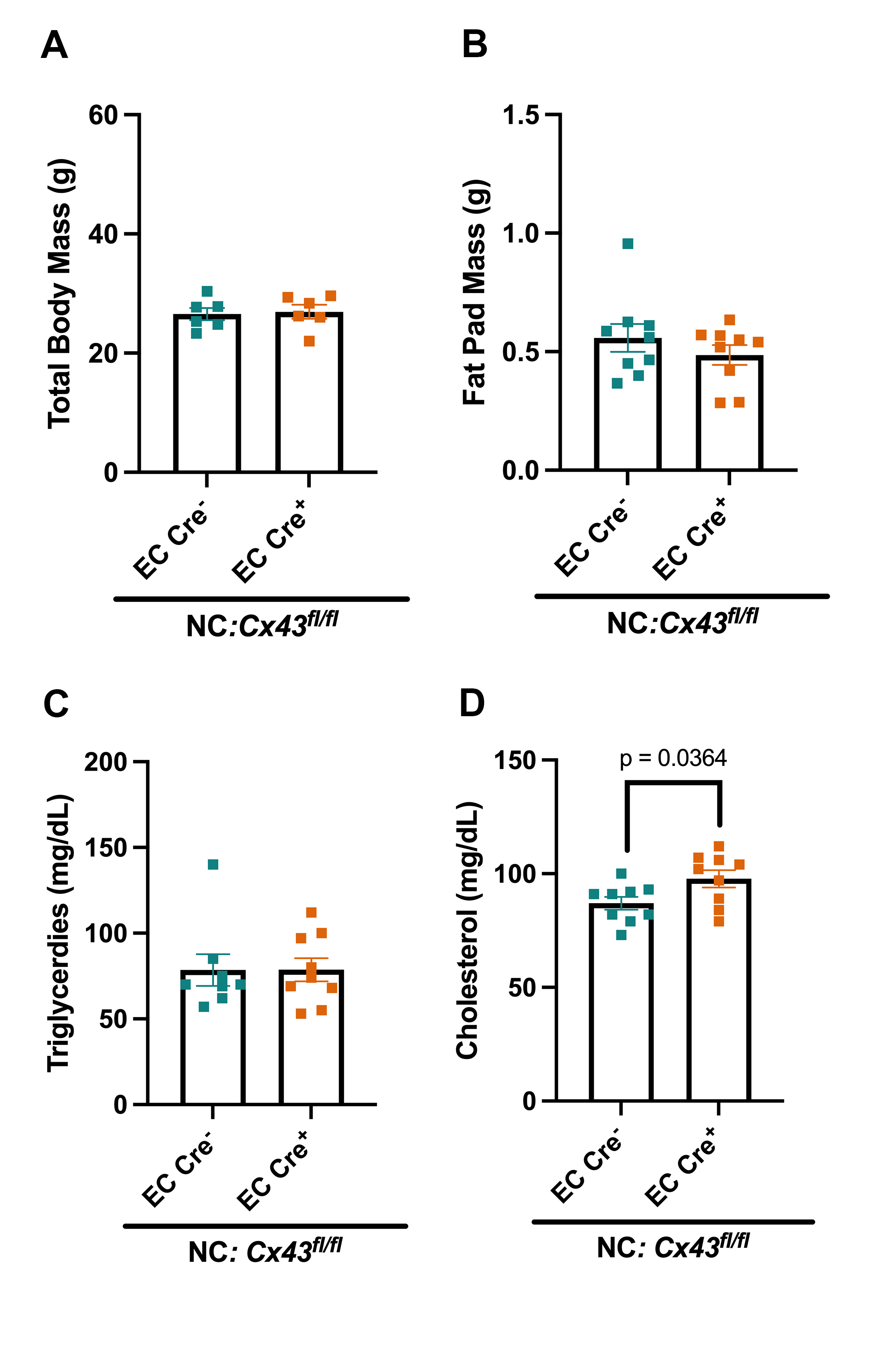
**

**Supplemental Figure 2: Deletion of EC Cx43 in normal chow fed male mice.** Normal chow (NC) male mice with (EC Cre^-^) and without (EC Cre^+^) EC Cx43. NC mice are aged matched with HFD mice. All mice between 22-25 weeks old. (A) Total body mass and (B) epididymal fat pad mass. Triglycerides (C) and Cholesterol (D). Student’s unpaired t-test.


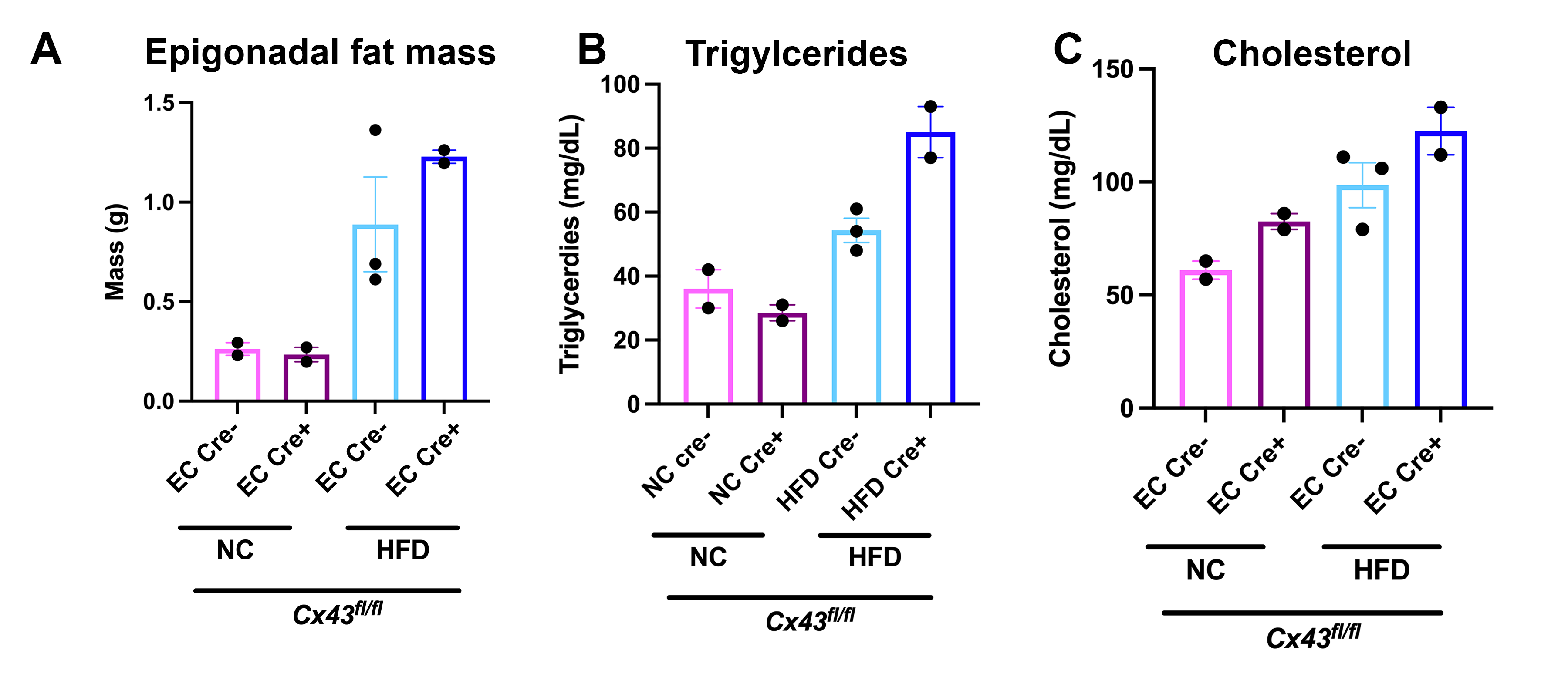


**Supplemental Figure 3: Metabolic parameters for from females.** NC and HFD female mice with (EC Cre^-^) and without (EC Cre^+^) EC Cx43. Epigonadal fat pad mass (A) Triglycerides (B) and Cholesterol (C) from female mice with or without EC Cx43 fed a NC or HFD for 12 weeks

**
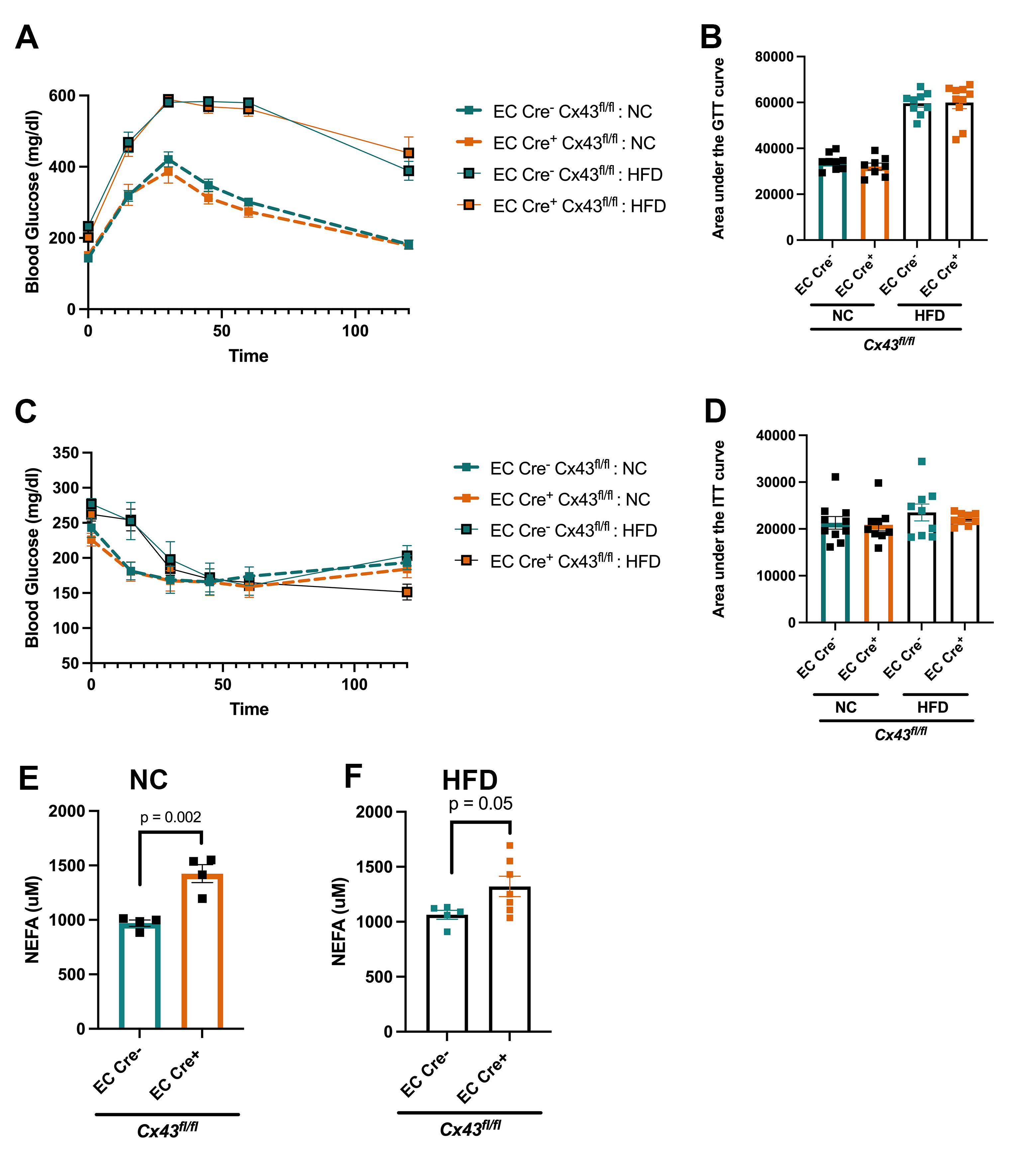
**

**Supplemental Figure 4: Metabolic analysis of EC Cx43 deletion.** High Fat diet (HFD) and Normal chow (NC) male mice with (EC Cre^-^) and without (EC Cre^+^) EC Cx43. NC mice are aged matched with HFD mice. All mice between 22-25 weeks old. (A) Glucose tolerance test with area under the curve quantified in (B). Insulin tolerance test, with area under the curve quantified in (D). Fasting non-esterified fatty acids (NEFA) levels in NC (E) and HFD (F) mice. P values from unpaired Student’s t-test.

**Supplemental Figure 5: Histological Analysis of Adipose and Liver.** HFD male mice with (EC Cre^-^) and without (EC Cre^+^) EC Cx43. H&E staining of Epigonadal fat pads, scale bar denotes 100µm (A) and Liver (B), scale bar denotes 200µm. (C) Oil Red O staining on cryo-sectioned livers, scale bar denotes 100µm. Area of oil Red O staining per image is quantified on the right. Four mice were used and three images per mouse were taken.
